# Joint Tuberculosis

**Published:** 1912-06

**Authors:** 


**Joint Tuberculosis.** By LEONARD W. ELY, M.D. Pp. xi, 243. Bristol: John Wright and Sons Ltd. 1911.

It is doubtful whether this book would have been written if a new edition of Sir Watson Cheyne's work on *Tuberculous Diseases of Bones and Joints* had appeared a little earlier, for the author, in his Preface, refers to this work as published some years ago, and he therefore considered there was some need for another book on the same subjects. But the second edition of Sir Watson Cheyne's book appeared a few months after Dr. Ely's was published. However, Dr. Ely's work is a valuable one, and gives us, in addition to his own observations, the views and practice of American surgeons, and those of other nationalities at the present time. He has studied the pathology of joint tuberculosis very thoroughly, and has observed much of its clinical aspect also. He first considers the pathology of joint tuberculosis in general, and also its symptoms, diagnosis and treatment, and then devotes chapters to the consideration of tuberculous disease of the spine and the various joints. The portion of the work devoted to the pathology of the disease seems to be the best illustrated, but there are reproductions of numerous skiagrams in the other part, though we think some more illustrations here would be an advantage.

The author explains why he has not more fully described operative technique. He says it is better described in works on general surgery. We hardly think so, and consider that in a work on joint tuberculosis all operative procedures recommended by the author, at any rate, should be fully described.

We notice that the surgical appointments held by the author are chiefly orthopædic. In America diseases of bones and joints are included under orthopædic surgery. This seems very remarkable to English surgeons, and we fail to see why orthopædic surgery, which is really the surgery of deformation, should include bones and joints. The argument seems to be that it should include them as they are organs of locomotion. As well might it include tendons and peripheral nerves. But orthopædic surgery does not deal with deformities of the organs of locomotion only; for instance, wry neck is a subject which would quite naturally come under such a division of surgery.

We cannot regard the account of tuberculous disease in the various joints as by any means complete. If we take the account of one of the most common forms of the disease—disease of the hip joint—we find several very important omissions. We do not expect the author to bring forward evidence which will convince his readers as to the common starting-point of the disease, for we know the great difficulty in procuring such evidence, but with regard to abscess formation we expect more than the somewhat vague statement that "abscess appears on the front of the thigh." We think it very doubtful that pus escapes from the joint by yielding of the capsule "anteriorly and below," as stated by the author. The method of formation of a "wandering acetabulum" is very imperfectly described, and its relation to true dislocation not clearly defined. Then when we turn to the "symptomatology" and diagnosis we fail to find any reference to the distinction, which those inexperienced so often fail to make, between absolute fixation from muscle spasm and true ankylosis. We are surprised also not to hear of the method which has been called by American surgeons "crowding the joint surfaces together" as a means of diagnosis. The author says it is unwise to attempt to elicit pain by striking the sole of the foot, "as it inflicts trauma," and therefore he has probably purposely omitted reference to crowding the joint surfaces together; but we think such a method may be a very valuable means of diagnosis in intra-osseous lesions, and cannot do any real harm. As well might we abstain from trying for movement in the joint for fear of "inflicting trauma."

We must say we are very dissatisfied with the author's recommendations as to the treatment of tuberculous hip joint disease. We do not like to hear of reduction of early deformity under an anæsthetic without, at any rate, a full trial of weight extension. Weight extension alone is quite sufficient in most cases, and under an anæsthetic the surgeon is very likely to employ more force than he should in dealing with a tuberculous joint. Of course, if there are not beds enough in the hospitals of the town in which Dr. Ely practices, there may be some excuse for the method he recommends. Most British surgeons have considered that it is not only important to apply a splint to a tuberculous hip so that it cannot be moved, but also to prevent weight bearing through the joint. Thomas's hip splint, used with a paten on the boot of the other foot, and crutches, does this. We are therefore much surprised to find the recommendation to treat these cases with a plaster spica, and let them get about freely on the affected limb, and to be told that this is the routine treatment in many hospitals in America at the present time. But the author says that weight bearing is not to be allowed in those cases in which a skiagram shows marked acetabular involvement, on account of the thinness of the bone at the acetabulum. We should feel quite unable to say that extensive disease did not exist because we did not see evidence of it in the skiagram. We suppose the author is afraid, if such disease exists, as he says the bone is thin, that if weight is borne on the limb the head will be forced through into the pelvis. But the bone forming the wall of the acetabulum is not thin. Whether there is much disease or little, either in the head or the acetabulum, we believe the removal of intra-osseous pressure by removing weight bearing from the limb is a very important element in the treatment of the case, and we therefore in this country prevent weight transmission through the limb. Another form of apparatus recommended by the author seems to us equally deficient in essential requirements. This is what he calls a "brace," and he describes and figures it. This apparatus prevents weight bearing, but it does not prevent movement at the joint, which should be the first object of our treatment. This imperfect apparatus is also supposed to make extension on the limb while the child is getting about. The apparatus seems to have been invented by those who held the erroneous view that fixation of a tuberculous joint caused ankylosis. Even the author admits how unsatisfactory it is as a means of making extension.

The author is not one of those surgeons who believe in excision as a valuable method of treatment even in suppurating cases. Indeed, he is opposed even to freely opening and scraping out the abscess, and says that all we must do is either to aspirate, inject, or possibly make small incisions, and cup after the manner of Klapp. He says all other operative treatment in children consists exclusively in amputation. We think if he practised a free opening and scraping out of abscesses, or if this failed, an excision, and maintained an aseptic wound, he would rarely have to amputate, which must always be looked on as an example of failure in treatment. He says "resection of the joint, done as a rule only in advanced cases, has a mortality of about 50 per cent." If he means that this fearful mortality occurs when done in advanced cases, it is even then a gross exaggeration; but if he means that this is the percentage of deaths of an operation which is usually performed in advanced cases, which seems the obvious meaning, that is even a greater exaggeration still. The mortality of the operation in cases of unopened abscess is very small, and even in cases with septic sinuses is by no means great.

The operative treatment of tuberculous disease of the hip in adults, recommended by the author, seems to us even more unsatisfactory than that suggested by him for children. His great aim, he tells us, is to deprive the joint of function, and avoid secondary infection. One would imagine from this that he aimed at ankylosis of the joint, and that the surgeon had to prevent secondary infection from auto-inoculation. What he does mean is that he advises excision of the head of the bone, and expects displacement of the cut section of the neck on to the dorsum ilei to give the joint rest, but in the end movement remains. He takes no trouble to remove as much tuberculous tissue from the joint as possible. He only says, "If one wishes, one may scrape the acetabulum." Most surgeons wisely do wish to scrape the acetabulum, and remove any sequestra found in it, and to cut or scrape away as much of the disease of the soft tissues of the joint as they can, as well as excise the diseased head of the femur. In tuberculous disease we wisely remove all the diseased tissue we can possibly get away, and generally nature is able to deal with the rest. It would be very unwise not to be as thorough as we can in removal of the tuberculous tissue from the joint because we cannot remove it completely. The author advises us to put up the limb after the operation in extension and slight abduction, and he says "this position crowds the stump of the neck of the femur against the pelvis." Now if it does so it must be against the diseased acetabulum, which the author has perhaps not even scraped, and thus he prevents that dislocation of the stump of the neck which he says it is his aim to produce. If he really wanted to produce dislocation at once, and placed his whole faith as to the efficiency of the operation in obtaining it, let him put up the limb not in abduction, but in adduction. But there is no need to crowd the stump of the neck against the innominate bone at all, and it will eventually get drawn up towards the dorsum; but the essential thing in the operation is the removal of the diseased tissue from the joint.

In describing the condition found on opening the joint when excision is performed, the author says the head of the femur may be found eroded. This is generally so. Then he also makes the remarkable statement that the head, denuded of its cartilage, may be found "adherent" to the acetabulum. Now this must be either fibrous or bony ankylosis, a condition which does not call for excision of the joint at all. We should very much regret cutting into a joint for purposes of excision, and finding that it had advanced so far towards recovery that such "adhesion" had occurred, unless after all active disease as well as deformity has to be considered. Of course, bony ankylosis with deformity may require operation, but this is a very different matter from operation in the active stage of the disease. We notice that for bony ankylosis the author says osteotomy of the neck of the femur may be performed; but there is very often no neck left when ankylosis occurs, as the part which was the neck may be united to the innominate bone, after complete destruction of head by caries. If we have to do an osteotomy for bony ankylosis following tuberculous hip-disease, the bone should be divided, or a wedge removed from the shaft, just below the level of the trochanter.

